# Was kommt dort durch die Luft geflogen …? Die Gefährdung des Menschen durch die Gifthaare des Eichenprozessionsspinners

**DOI:** 10.1007/s12326-021-00421-1

**Published:** 2021-02-18

**Authors:** Harald Maier

**Affiliations:** Universitätsklinik für Dermatologie, Medizinische Universität Wien, Währinger Gürtel 18–20, 1090 Wien, Österreich

**Keywords:** Eichenprozessionsspinner, Lepidopterismus, Raupendermatitis, Brennhaare (Setae), Urbaner Grünbereich, Oak processionary moth (caterpillar), Lepidoperism, Caterpillar dermatitis, Urticating hairs (setae), Urban green

## Abstract

Die Larven des 4. bis 6. Stadiums des Eichenprozessionspinners sind mit Brennhärchen (Setae) bewehrt, welche bei Mensch und Tier heftige entzündliche Haut- und Schleimhautreaktionen (Lepidopterismus) hervorrufen. Die Übertragung erfolgt sehr häufig aerogen, oder durch direkten Kontakt. Durch den Befall von Bäumen im urbanen Grünbereich ist die Fallzahl – vor allem in Jahren mit Massenvermehrung des Forstparasiten – sehr groß. Da die Dunkelziffer sehr hoch ist, sollten Ärzte und Ärztinnen bei Patienten mit juckenden, asymmetrisch verteilten papulösen oder urtikariellen Exanthemen an die Möglichkeit des Vorliegens einer Raupendermatitis denken. Meiden der Befallsgebiete bzw. Tragen von persönlicher Schutzkleidung bei Außenarbeitern, stellen die besten präventiven Maßnahmen dar.

## Abstract (en)

Fourth to sixth instar larvae of the oak processionary moth protect themselves against predators by urticating hairs (setae). Setae may cause inflammatory reactions on skin as well as on mucous membranes (lepidopterism), both in humans and animals. People may get in touch with setae by direct contact or – more frequently – airborne. Frequency of lepidopterism is high due to the fact that the oak processionary moth infests the urban green, especially in years with mass gradation. Lepidopterismus, however, is under-diagnosed. In seasons of toxic larvae, doctors should therefore consider lepidopterism a differential diagnosis in patients with asymmetrically distributed, itchy papular rash or wheal formation. Preventive measures are avoidance of infested areas and use of protective equipment by outdoor workers at risk.

Durch die COVID-19-Pandemie ist der Begriff „Aerosol“ als potenzieller Vektor der Übertragung von Noxen – in diesem Fall des SARS-CoV-2-Virus – den meisten Menschen ein Begriff. Auch dieser Artikel befasst sich mit einer biologischen Noxe, welche (vielfach) aerogen übertragen wird, worauf auch der Titel meines Artikels Bezug nimmt [1]: den Gifthaaren (Setae) des Eichenprozessionsspinners (Thaumetopoea processionea Linné; EPS; [2, 3]). Obwohl das Ausmaß der Bedrohung weit hinter der Bedrohungslage durch die Viruspandemie zurückliegt, kann man einige Parallelen erkennen.

Wie bei COVID-19 handelt es sich um eine biologische Noxe, im Falle der Setae allerdings um pfeilförmige Chitinhärchen (Abb. 1) mit einer brisanten Füllung aus dem Eiweißgift Thaumetopoein (Tha p2; [4]), welches als Gift und/oder Allergen wirkt, sowie noch nicht näher definierten Begleitproteinen. Wie bei COVID-19 gibt es viele Unklarheiten zur sinnvollen Prävention, und die Zunahme ihrer Bedeutung für den Menschen verdanken beide Phänomene einem menschlichen Fehlverhalten. Während das SARS-CoV-2-Virus, einer Hypothese zufolge, durch einen zu engen Kontakt mit bestimmten Tieren auf den Menschen übersprang, zählt der EPS zu den Gewinnern der globalen Erwärmung [5–8]. In einer gewissen Weise zeigen uns beide Gesundheitsprobleme mehr oder weniger deutlich die Dekadenz unseres *modernen* Lebensstils auf.

**Figure 1:**
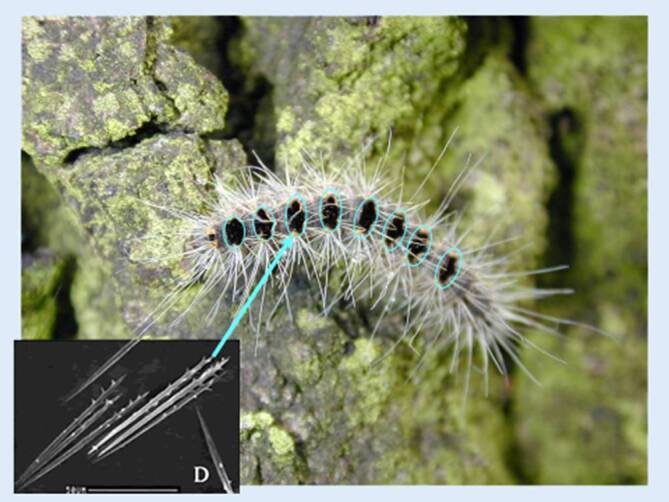

Der EPS ist ein endemischer Forstschädling (Abb. 2 und 3), dessen Verbreitungsgebiet von Kleinasien im Osten bis an die Kanalküste im Westen reicht [9]. Einzelne umschriebene Verbreitungsgebiete finden sich in Südengland und im Großraum London, wohin der EPS durch befallene Eichensetzlinge aus den Niederlanden gekommen ist. Der EPS frisst nur an bestimmten Eichenarten und richtet dabei einen beträchtlichen forstwirtschaftlichen Schaden an [10, 11].

**Figure 2:**
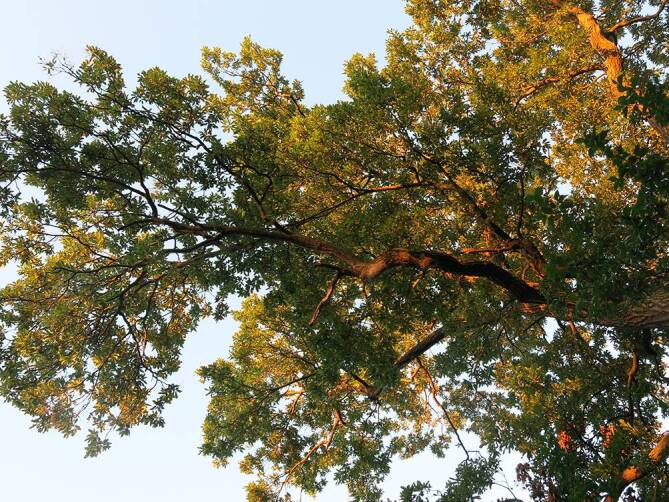

**Figure 3:**
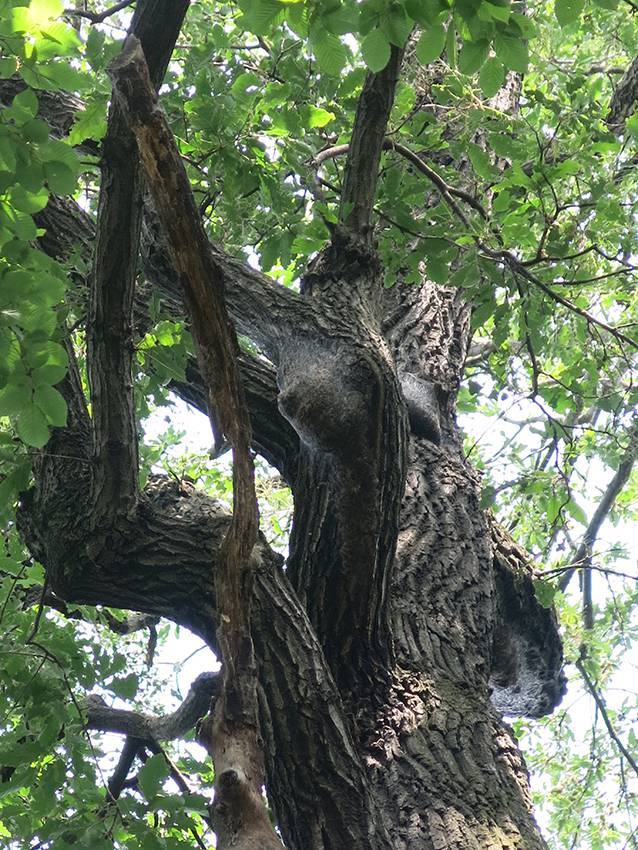

Eine Bedrohung für die menschliche Gesundheit wird der Forstschädling aufgrund seines besonderen Verhaltens. Er befällt Eichen in Randlage, um Lichtungen und entlang von Wegen, beziehungsweise einzeln stehende Bäume und Baumgruppen. In Wien, das sich ja im Bereich eines Buchen-Eichen-Misch(ur)waldes entwickelte, dringt der EPS daher am Stadtrand in Wohngebiete vor (z. B. Pötzleinsdorf, Heuberg, Schafberg) und verbreitet sich in Naherholungsgebieten der Stadt, wie Schönbrunner Schlosspark, Garten des Palais Liechtenstein, Unteres Belvedere, Lainzer Tiergarten, Donauinsel, Laaerberg mit Böhmischem Prater u. a. Dadurch treten Erscheinungen der Raupenkrankheit (Lepidopterismus) nicht nur berufsbedingt bei Forstarbeitern und Gärtnern auf, sondern auch bei Anwohnern oder Besuchern dieser Befallsgebiete [2].

Der Eichenprozessionsspinner ist ein endemischer Forstschädling

Nicht selten sind ganze Kindergartengruppen oder Schulklassen betroffen, die ihre Ausflüge in diese Naherholungsräume unternehmen [12, 13]. Da auch die urbanen Grünbereiche der deutschen Bundeshauptstadt Berlin in den vergangenen Jahren massiv von der EPS-Plage betroffen waren, beauftragte mich das Deutsche Umweltbundesamt, eine ausgedehnte Untersuchung zu offenen Fragen über die Gefährdung des Menschen durch die Gifthaare des EPS durchzuführen. Der Abschlussbericht des Forschungsprojekts wird gerade fertiggestellt (Projektnummer FKZ 3712 62 203).

## Zoologie

Zoologisch gesehen handelt es sich beim EPS um einen Nachtfalter, der sich über 5 bis 6 Raupenstadien und ein Puppenstadium entwickelt [14]. Die Falter schlüpfen Mitte Juli und leben nur 1 bis 2 Tage, um sich zu paaren und die Eier in bestens getarnten Gelegen in den sonnenbeschienenen Baumkronen abzulegen. Die Eigelege überwintern und aus ihnen schlüpfen ab Mitte April bis Mai die Eiraupen. Für die Pathogenese der Raupenkrankheit sind in erster Linie das 4. bis 6. Raupenstadium (Abb. 1) von Bedeutung, da die Tiere zur Abwehr von Fressfeinden an den Rückensegmenten dichte Bürsten von Gifthärchen entwickeln (Abb. 1).

Im letzten Raupenstadium ist jedes Individuum mit 500.000 bis 600.000 solcher Setae bewehrt. Diese brechen an Sollbruchstellen bei der leisesten Berührung ab und werden aufgrund ihrer idealen Flugeigenschaften [15] mehrere Hundert Meter weit mit dem Wind vertragen. Damit nicht genug, bleiben die Härchen bis zu 10 Jahre in der Umwelt aktiv, da sie UV- und hitzeresistent sind.

Die Härchen bleiben bis zu 10 Jahre in der Umwelt aktiv, da sie UV- und hitzeresistent sind

**Figure 4:**
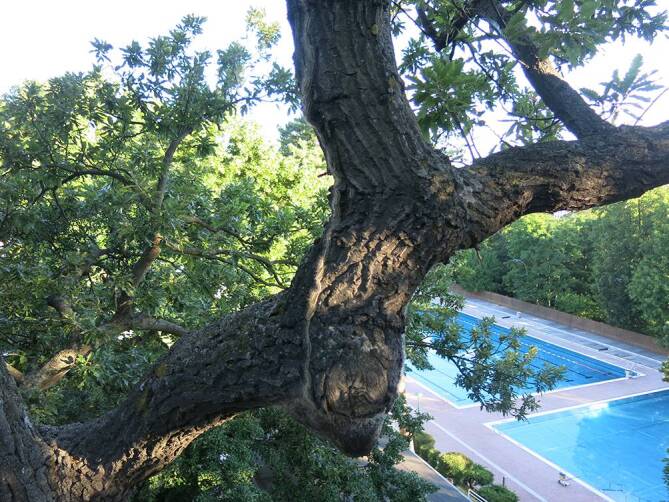

**Figure 5:**
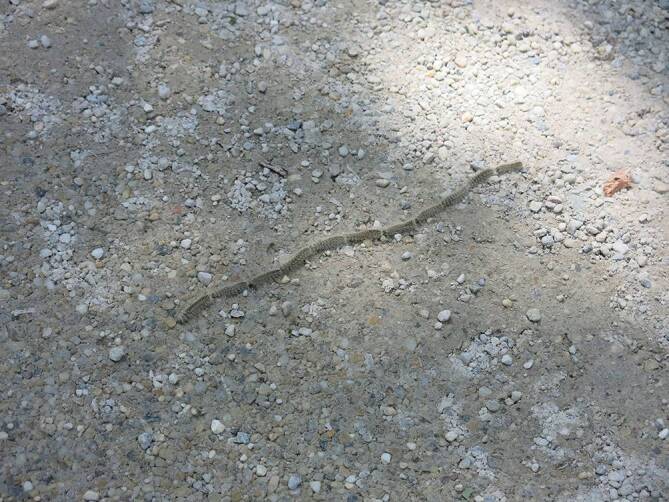

**Figure 6:**
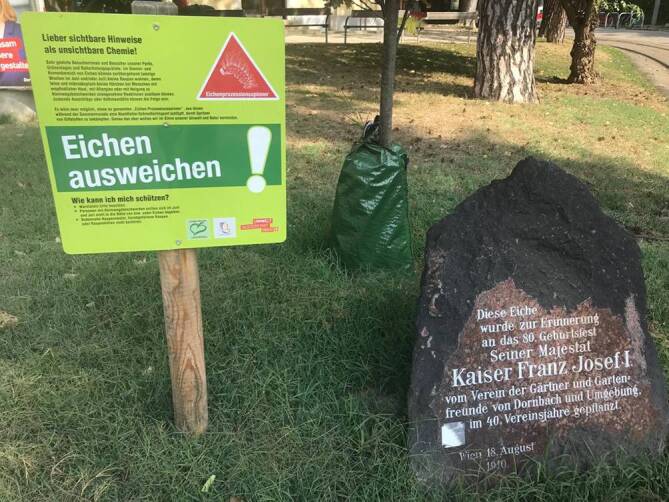

Damit gibt es auch Fälle von Lepidopterismus außerhalb der Giftraupenphase von Mitte Mai bis Ende Juni, z. B. durch Aufwirbeln der Haare bei diversen Gartenarbeiten und durch Kontakt mit kontaminiertem Brennholz aus befallenen Forsten. Dem Raupenstadium verdankt die Art auch ihre Bezeichnung. Die Tiere ziehen in ganzen Prozessionen zu den Baumwipfeln, um dort in der Nacht an den Eichenblättern zu fressen. Von dort kehren sie in geschlossener Formation frühmorgens wieder in ihre Nester zurück (Abb. 4). Bei Nahrungsmangel oder Überpopulation kann man sie auch am Boden antreffen, wo sie zu einem potenziellen neuen Wirtsbaum prozessionieren (Abb. 5). Ein besonderes Phänomen, dessen Ursachen noch nicht restlos aufgeklärt sind, ist die Massenvermehrung in unregelmäßigen Abständen. Dabei kommt es zu einer enormen Vermehrung, die den EPS zu einer richtigen Landplage werden lassen. Dies sind auch Zeiten, in denen der Zutritt zu besonders befallenen Naherholungsgebieten gesperrt ist (Abb. 6).

## Die Raupendermatitis

**Figure 7:**
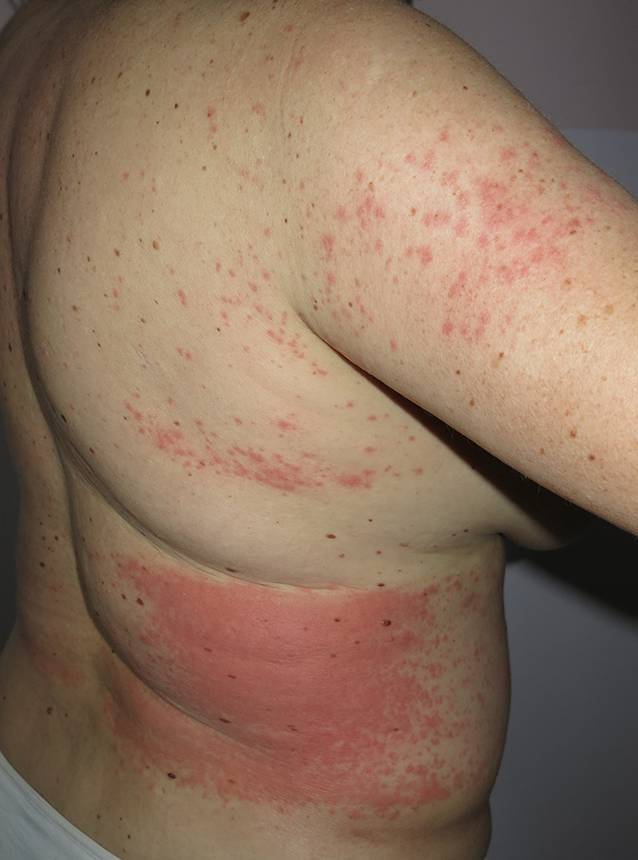

**Figure 8:**
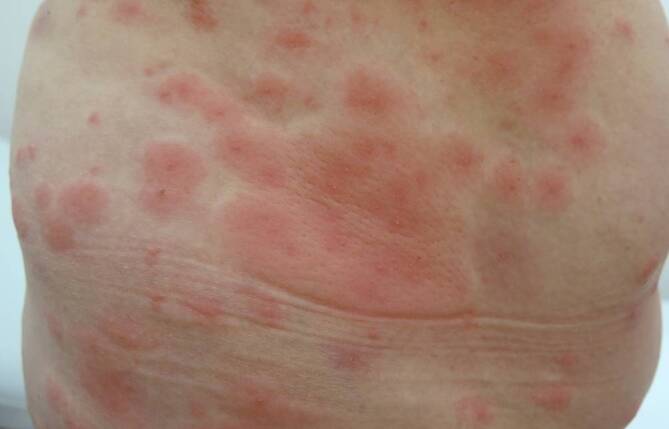

Wodurch macht sich nun ein Kontakt mit Setae bemerkbar? Die Gifthärchen dringen in Haut und Schleimhäute ein und lösen dort umschriebene Entzündungsreaktionen aus. An der Haut bezeichnet man dies als Raupendermatitis, die in drei Varianten vorkommt [2]. Die häufigste Erscheinungsform ist eine irritativ-toxische (klein-papulöse) Form (Abb. 7), gefolgt von einer großknotigen, über mehrere Wochen persistierenden Variante. Die Kontakturtikaria stellt die seltenste dermatologische Erscheinungsform dar (Abb. 8).

Strittig ist noch immer die Antwort auf die Frage, ob Setae des EPS auch anaphylaktische Reaktionen auslösen können. In den von uns erhobenen Daten fanden sich dafür allerdings keine Hinweise. Damit unterscheidet sich die Pathogenese des EPS-induzierten Lepidopterismus von dem klinischen Spektrum, welches durch Kontakt mit Setae des Pinienprozessionsspinners (PPS) – eines mediterranen Verwandten des EPS – ausgelöst wird. Die Literatur berichtet im Zusammenhang mit PPS immer wieder von anaphylaktischen Reaktionen [16].

Für Österreich spielt der PPS eine untergeordnete Rolle, obwohl seit einigen Jahren vom Kanaltal eine Ausbreitung auf das südliche Bundesgebiet zu beobachten ist [17]. Andere Organe, die betroffen sein können, sind das äußere Auge und der obere Respirationstrakt. Ob das Bronchialsystem des Menschen ebenso betroffen ist, wie das von Tieren (z. B. Pferde), soll eine neue Untersuchung zeigen.

## Therapie

Keine der beschriebenen dermatologischen Manifestationen ist pathognomonisch, deshalb ist es das Wichtigste, „daran zu denken“, wenn ein Patient mit einem heftig juckenden Ausschlag zur Giftraupensaison in der Ordination erscheint und noch dazu berichtet, in einem Endemiegebiet unterwegs gewesen zu sein. Die Behandlung erfolgt symptomatisch mit Antihistaminika und topischen Glukokortikoid-Mischungen. Nur selten ist der Einsatz von systemischem Kortison indiziert.

Der Nachweis der Setae ist schwierig, da die Härchen sich in der H.E.-Färbung nicht spezifisch anfärben. Spezialfärbungen sind hilfreich, und vielfach finden sich auch Setae auf dem Tixo-Abklatsch von Haut oder Textilien. Da auch die Kenntnis über unsere haarigen Mitbewohner des Wiener Stadtgebietes gering ist, wundert die hohe Dunkelziffer an Lepidopterismus-Fällen nicht. Die wichtigsten Eckdaten fasst die Tab. 1 zusammen.

**Table 1:** 

Art/Gattung/Familie/Ordnung	*Thaumetopoea processionea* Linné (Eichenprozessionsspinner, „oak processionary moth“)/Thaumetopoea/Zahnspinner/Schmetterlinge (Lepidoptera)	–
Biologie	Forstparasit an *Quercus cerris *und *Quercus robur*	–
	5–6 Larvenstadien, Puppenstadium, adulte Schmetterlinge, Eigelege	
Ökologie	Gewinner des Klimawandels	*Massengradation* *=* *massenhafte Vermehrung des EPS durch das günstige Zusammenspiel verschiedener Umweltfaktoren*
	Fehlen natürlicher Feinde	
	Milde Winter, gleichmäßige Frühjahrstemperaturen, geringe Niederschlagsmengen im Frühjahr, Laubaustrieb synchron mit Schlüpfen der Eilarven	
Kausale Pathogenese	Setae = pfeilförmige Chitingebilde mit Widerhaken (Spikulae) und einem geschlossenen Hohlraum, Inhalt aus Tha p2 und n. d. Begleitstoffen	*500.000 bis 600.000 Setae pro Larve im 6. Stadium, verteilt auf die 8 letzten Rückensegmente*
Formale Pathogenese	Direkter Kontakt	– Pruritus – Irritativ-toxische papulöse Dermatitis – Persistierende noduläre Dermatitis – Kontakturtikaria *(anaphylaktische Reaktionen fanden sich weder anamnestisch noch klinisch bei keinem Patienten unseres Studienkollektivs)*
	Aerogene Übertragung – Raupendermatitis – Keratokonjunktivitis – Reizung des oberen Respirationstrakts – Schwindelgefühl, Benommenheit – Lungenbeteiligung? – Anaphylaktische Reaktion??	
Risikofaktoren	Außenberufe in Befallsgebieten	–
	Anrainer von Befallsgebieten	
	Freizeitaktivitäten in Befallsgebieten	
	Windige Wetterlagen	
Diagnosekriterien	Anfang Mai bis Ende Juni	*Eine Sichtung von EPS ist aufgrund der häufigen, aerogenen Übertragung zur Diagnosestellung nicht erforderlich*
	Meist asymmetrisch verteilte, dicht stehende, heftig juckende entzündliche Knötchen/Knoten, oder Quaddeln	
	Anamnestisch Aufenthalt in einem Risikogebiet	
Prävention	Gesetzliche Regelung der Verantwortung für Präventivmaßnahmen [19]	*Bt: massiver Kollateralschaden an der heimischen Schmetterlingspopulation*
	Betretungsverbot	
	Persönliche Schutzkleidung (Pestizide sind in Österreich zur Bekämpfung des EPS verboten)	
	Bazillus thuringensis (Bt)	
	Predatoren	
	Mechanische Entfernung der Nester	
	(Ausforstung der parasitierten Bäume)	
Therapie	Antihistaminika	–
	Glukokortikosteroidexterna	
	Systemische Glukokortikosteroide	

## Prävention

Weit wichtiger als die Behandlung ist die Prävention. Neben gezielten (biologischen) Bekämpfungsmaßnahmen ist das Vermeiden des Aufenthalts in befallenen Gebieten die sinnvollste Maßnahme. Dafür bedürfte es gezielter, aktualisierter Warnhinweise über die aktuelle Befallssituation.

Bei Freizeitaktivitäten ist dies relativ einfach zu bewerkstelligen: Man joggt eben nicht auf den Wegen im Schönbrunner Schlosspark nahe dem Hohenbergtor, welche durch stark befallene Parkareale führen, und ignoriert die dort angebrachten Absperrbänder! Im Fall potenzieller beruflicher Exposition ist Prävention durch Vermeidung ungleich schwieriger. Hier muss der Arbeitgeber seinen Arbeitnehmern entsprechende persönliche Schutzausrüstung zur Verfügung stellen.

## Die Raupensaison

Möge dieser Artikel über die Gefahren durch diese wehrhaften Zeitgenossen zur Verbesserung der Diagnostik beitragen, da die nächste Raupensaison ja wieder bevorsteht.

Wie stark die EPS-Population 2021 ausfallen wird, ist schwer vorherzusagen. Forstentomologische Methoden (Falterfänge, Eigelege-Zählungen) ermöglichen eine gewisse Orientierung. Allerdings ist das Überleben der Raupen nicht unwesentlich von diversen Umweltfaktoren abhängig.

Neben milden Wintern begünstigt das Fehlen von Spätfrösten in relativ niederschlagsarmen Frühjahren das Überleben der Gifttiere. Viel zu wenig ins Kalkül gezogen wird auch die Synchronie von Laubaustrieb an den Wirtsbäumen und Schlüpfen der Eiraupen [18]. Zumindest der milde Winter 2020/21 verheißt für die kommende EPS-Saison nichts Gutes, sollte es nicht noch zu Phasen mit tiefen Temperaturen kommen.
